# Venetoclax ex vivo functional profiling predicts improved progression-free survival

**DOI:** 10.1038/s41408-022-00710-9

**Published:** 2022-08-04

**Authors:** Vikas A. Gupta, Shannon M. Matulis, Benjamin G. Barwick, R. Devin Bog, Conrad W. Shebelut, Mala Shanmugam, Paola Neri, Nizar J. Bahlis, Madhav V. Dhodapkar, Leonard T. Heffner, Craig C. Hofmeister, Nisha S. Joseph, Sagar Lonial, Jonathan L. Kaufman, David L. Jaye, Ajay K. Nooka, Lawrence H. Boise

**Affiliations:** 1 Department of Hematology and Medical Oncology, Winship Cancer Institute of Emory University, Emory University School of Medicine, Atlanta, GA USA; 2 Department of Pathology and Laboratory Medicine, Winship Cancer Institute of Emory University, Emory University School of Medicine, Atlanta, GA USA; 3 Arnie Charbonneau Cancer Research Institute, University of Calgary, Calgary, AB Canada

**Keywords:** Targeted therapies, Myeloma

**Dear Editor,**

In multiple myeloma, the t(11;14) translocation enriches for patients likely to respond to the Bcl2 inhibitor venetoclax. In this group of patients, 40% respond to single-agent venetoclax while up to 60% respond to the combination of venetoclax and dexamethasone [1, 2]. We have previously demonstrated that ex vivo functional profiling of venetoclax sensitivity can more accurately identify these venetoclax-responsive patients [3]. Here we report updated data on a larger cohort of patients who underwent ex vivo testing and were subsequently treated with venetoclax. We demonstrate that this 24-hour functional assay can rapidly predict patient responses to venetoclax that translate into improved progression-free survival (PFS).

Between April 2014 and June 2020, we performed a 24 h ex vivo apoptosis analysis on 33 patients who went on to receive venetoclax therapy including 14 patients from our previous report [3]. One patient with non-secretory disease and four patients that received venetoclax in combination with daratumumab or carfilzomib were excluded. Nineteen patients were classified as sensitive to venetoclax based on an IC50 of <100 nM while nine had an IC50 greater than 100 nM and were considered resistant (supplemental table 1, supplemental Fig. 1). The patient characteristics were well balanced between the two groups (supplemental Table 2). All patients were positive for t(11;14) with the exception of two patients in the sensitive group. Both sensitive (range 1–7) and resistant (range 2–6) patients experienced a median of three prior lines of therapy. Based on pre-clinical data indicating synergy between venetoclax and dexamethasone [4] and data from the venetoclax plus dexamethasone phase 1/2 expansion [2], 63.2% of sensitive patients received concurrent dexamethasone as did 77.8% of resistant patients.

The overall response rate in sensitive patients based on the ex vivo assay was 89.5% vs. 44% in the resistant group (*P* = 0.032, Fig. 1A). In the sensitive group, 58% of patients achieved a VGPR or better compared to 33% in the resistant group. Of the two patients in the sensitive group that did not achieve a PR, one patient had a 35% reduction in paraprotein and stable disease for >319 days, for a clinical benefit rate of 95% vs. 44% in the resistant group. Median progression-free survival was 23.2 months in the sensitive group compared to 4.8 months in the resistant group (*P* = 0.0205, HR 0.36, 95% CI 0.11–1.18, Fig. 1B). Median overall survival was not reached in the sensitive group and was 32.6 months in the resistant group (*P* = 0.2084, HR 0.33, 95% CI 0.07–1.65, Fig. 1C). A receiver operating characteristic curve analysis for the ex vivo assay revealed an area under the curve of 0.8231 (*P* = 0.0117, Fig. 1D).

**Fig. 1:**
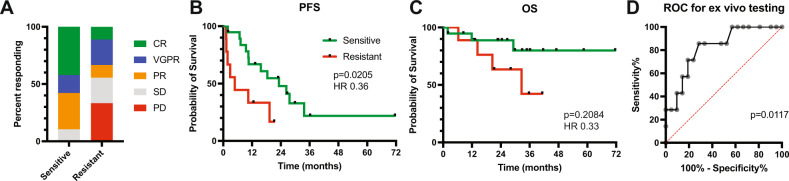
Patient outcomes based on ex vivo venetoclax testing. Pre-treatment bone marrow aspirates were tested ex vivo for venetoclax sensitivity as described previously [3]. Briefly, buffy coat cells were treated with increasing concentrations of venetoclax for 24 h and then assessed for apoptosis by annexin V staining to determine an IC50. An IC50 of less than 100 nM was considered sensitive, while an IC50 of greater than 100 nM was considered resistant. **A** Overall response rate for patients with sensitive vs. resistant ex vivo testing. **B** PFS and **C** OS of sensitive and resistant patients. **D** Receiver operating curve for ex vivo testing.

Particularly noteworthy are the two non-t(11;14) patients in our cohort, MM109 and MM116, both of whom were sensitive to venetoclax on ex vivo testing and went on to respond to venetoclax therapy. MM109 obtained a VGPR after lenalidomide induction, melphalan 140 mg/m^2^ and autologous stem cell transplant, and lenalidomide maintenance. Upon relapse, he started single-agent venetoclax and has been in a sustained PR for almost three years. MM116 was primary refractory to dara-RVD induction and subsequently received two cycles of VDT-PACE followed by high dose melphalan and autotransplant, achieving a VGPR. He was then started on venetoclax and deepened his response to a sCR. Although both patients lacked t(11;14), MM109 possessed an amplification of CCND1 with up to 20 copies on FISH, and MM116 was positive for trisomy of chromosome 11. Seven of the 9 resistant patients, including all 4 of the responders in the resistant group, received dexamethasone as a second agent in combination with venetoclax which may account for the relatively high response rate in the resistant group. We tested the combination of venetoclax and dexamethasone ex vivo in three of those four patients, two of which demonstrated significant sensitization to venetoclax (MM95 and MM156-3; supplemental Fig. 2).

Our results compare favorably to the phase 1/2 study of venetoclax plus dexamethasone which treated 51 patients with t(11;14) [2]. The overall response rate in that non-selected population was 60% in the phase 1 portion (*n* = 20) and 48% in the phase 2 portion (*n* = 31). The median time to progression was 12.4 months and 10.8 months, respectively. However, a direct comparison of our results is limited by differences in the patient population. Patients in the phase 2 portion were in general more heavily pre-treated with at least two lines of therapy (median of 5). All were refractory to a proteasome inhibitor, and most were refractory to an immunomodulatory agent and daratumumab. In our patient population, only 46% were refractory to daratumumab prior to treatment with venetoclax.

We have previously demonstrated that increased binding of pro-apoptotic proteins to BCL2 also correlates with response to BCL2 inhibitors, however, such complex studies are challenging to implement in clinical laboratories [5, 6]. To compare our ex vivo analysis to BCL2 protein expression, we measured BCL2 by IHC on FFPE slides obtained immediately prior to the initiation of venetoclax for 25 of the 28 ex vivo tested samples. Seven samples scored as low, 4 as intermediate, and 14 as high (Fig. 2A). ORR was 89% in the combined intermediate/high BCL2 group and 29% in the low group. PFS was 26.3 months compared to 4.8 months, respectively (*P* = 0.0154, HR 0.32, 95% CI 0.09–1.23, Fig. 2B). Notably, the two resistant samples that were either not sensitized to dexamethasone (MM129) or not tested with dexamethasone (MM182), but achieved a response to venetoclax, both had intermediate or high BCL2 IHC (supplemental Fig. 2). Thus, IHC performed similarly to our ex vivo testing. We then examined the combination of IHC and ex vivo testing. Results from the two assays were concordant in 17 cases, 13 of which were sensitive by ex vivo testing with intermediate/high BCL2 and 4 of which were ex vivo resistant and BCL2 low. In this group of patients, the combined assay had a sensitivity of 93% and specificity of 100% (*P* = 0.059). Although challenges remain with translating ex vivo testing to routine use, shortcomings of IHC include greater turnaround time as well increased variability and subjectivity in scoring. To improve the accuracy of our ex vivo assay we are now consistently co-testing with dexamethasone, which may have contributed to the patient responses seen in some of our resistant samples. Steps to address feasibility include potentially automating the assay in a 96-well format as well as further reducing the time needed to obtain results. Indeed, we have piloted a 3 h assay with similar results to the 24 h assay described here.

**Fig. 2:**
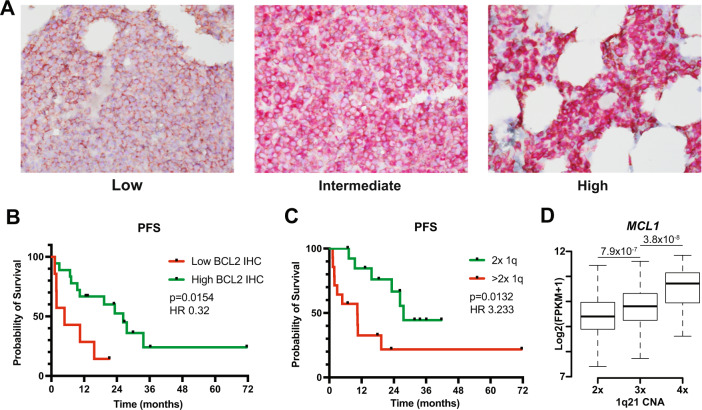
Patient outcomes based on BCL2 IHC. Pre-treatment bone marrow biopsies were co-stained by IHC for BCL2 and CD138 to identify plasma cells. Samples were grouped into low and intermediate/high categories by a pathologist blinded to clinical correlates. **A** Representative images of bone marrow biopsies with low, intermediate, and high BCL2 (red) staining in CD138 (brown) plasma cells. **B** PFS of patients with low vs intermediate/high BCL2 staining in plasma cells. **C** PFS of patients treated with venetoclax having either 2 or greater than two copies of 1q. **D** Copy Number Alterations (CNA) at chromosome 1q21 and MCL1 expression in 670 Newly Diagnosed Multiple Myeloma (NDMM) cases from the CoMMpass study. (2×, N = 430; 3×, *N* = 197; 4× *N* = 43). *P* values computed using a *t* test are denoted on top.

BCL2 expression by RNA or IHC has also been studied in venetoclax trials. In the phase 1 study of venetoclax, the ratio of BCL2 to MCL1 was higher in responders compared to non-responders [1], while BCL2 expression was higher in responders on the venetoclax plus dexamethasone study as well as the phase 1 study of venetoclax plus bortezomib [2, 7]. However, in all cases, there is a significant amount of overlap between responders and non-responders. We have made similar observations in ex vivo tested patient samples and cell lines [8]. In the cohort reported here, BCL2 RNA expression did not correlate with PFS or IHC score in the subset of 16 patients for whom we had RNAseq data (supplemental Fig. 3). Expression of other BCL2 family members, both pro- and anti-apoptotic including BCL2L1 (Bcl-xL), MCL1, BCL2L11 (Bim), BBC3 (Puma), PMAIP1 (Noxa), BID, BAK1, and BAX, did not differ between responders vs. non-responder patients or sensitive vs. resistant samples (supplemental Fig. 4A, B). We have also reported on the use of B-cell markers to predict venetoclax sensitivity in myeloma, however, clinical flow data were available for only CD20, which did not correlate with PFS (supplemental Fig. 5), consistent with our previous results using a panel of B-cell and plasma cell markers [8].

In our cohort, a gain of 1q21 was associated with worse progression-free survival (HR 3.23, 95% CI 1.15–9.09, *P* = 0.0132; Fig. 2C). Five (26%) sensitive and nine (100%) resistant patients had gained at least 1 copy of 1q (*p* = 0.001). The 1q21 segment contains MCL1, another anti-apoptotic BCL2 family member that may be a source of resistance to venetoclax [9], and 1q21 copy number correlates with MCL1 expression in the CoMMpass data set (Fig. 2D). The IL6 receptor, which we have previously demonstrated to mediate resistance to venetoclax through IL6 signaling and increased MCL1 expression, is also present in 1q21 [5]. Gain of 1q21 has been reported to result in early progression with other treatments and increased sensitivity to the MCL1 inhibitor S63845 [10, 11]. Together these data suggest that increased MCL1 expression from a gain of this locus may mediate a reciprocal dependence on BCL2 vs MCL1 and may contribute to venetoclax resistance even in the presence of t(11;14). Nevertheless, 56% of patients with a gain of 1q21 responded to venetoclax (compared to 100% of patients without 1q21 gain) and the duration of response was not significantly different between the two groups (*p* = 0.58), suggesting that 1q21 alone should not be used to exclude patients from receiving venetoclax.

In light of the potential for increased toxicity and mortality with venetoclax combinations, identifying patients likely to benefit from single-agent venetoclax takes on added importance. Although t(11;14) FISH and BCL2 IHC performed well at predicting responses to venetoclax, our assay can be completed in 24 h compared to days for FISH and IHC, thus allowing more rapid clinical decision making. We have also previously demonstrated that there are a group of non-t(11;14) patients expression B-cell markers [8] that would be excluded if venetoclax was limited to t(11;14) patients. It is in this larger population of patients initially selected based on the presence of t(11;14) or B-cell markers where ex vivo testing may have the greatest utility and will be the focus of future studies. Ex vivo testing as performed here, and similar assays such as BH3 profiling, bypass the limitations of measuring RNA and protein expression or protein-protein interactions and ultimately integrate numerous inputs to measure cell death as the final downstream outcome [12]. We demonstrate that results from our 24-hour ex vivo functional assay can rapidly and effectively predict responses to venetoclax that correlate with improved outcomes and may therefore serve as a biomarker to guide therapy decisions.

## Supplementary information

Supplemental Methods and Figures

Reproducibility checklist

## Data Availability

Emory patients sample RNAseq data are deposited in GEO and publicly available (GSE167969). CoMMpass RNAseq data is deposited in dbGAP (accession phs000748).
