# Supplementary material for: Venetoclax ex vivo functional profiling predicts improved progression-free survival
Source: Blood Cancer J. 2022 Aug 4;12(8):115. doi: 10.1038/s41408-022-00710-9 (PMC9352682; doi:10.1038/s41408-022-00710-9)
Supplement: Supplementary file 1 — Supplemental Methods and Figures [file 41408_2022_710_MOESM1_ESM.pdf]

Supplementary Materials

Methods

Reagents

Propidium iodide (PI) and Dexamethasone (Dex) were purchased from Sigma-Aldrich (St Louis, MO); Venetoclax was provided by AbbVie; Annexin-V–fluorescein isothiocyanate (FITC) was purchased from Biovision (Palo Alto, CA); Annexin-V-Pacific Blue was purchased from ThermoFisher Scientific (Waltham, MA); anti-CD38-phyocerythrin (PE, 345806), anti-CD45-allophycocyanin-cyanine 7 (APC-Cy7, 557833), anti-CD138-FITC, and anti-CD20-peridinin-chlorophyll-protein-cyanine 5.5 (PerCP-Cy5.5, 332781) antibodies were purchased from BD Biosciences (San Jose, CA); multi-epitope CD38-FITC was purchased from American Laboratory Products Company (ALPCO, Salem, NH).

Patient Sample Processing

All samples were collected from patients that had signed informed consent following an Emory University Institutional Review Board-approved protocol. Bone marrow aspirates from consenting myeloma patients were diluted to 25 mL with PBS, filtered and underlaid with lymphocyte separation medium (Mediatech, Manassas, VA). The buffy coat was collected, washed with PBS, re-suspended in RPMI-1640 supplemented with 10% heat- inactivated fetal bovine serum, 100 U/mL penicillin, 100 g/mL streptomycin, and 2 mM L-glutamine (Cellgro, Mediatech, Herndon, VA). Cells were stained with anti-

CD38-PE, anti-CD45-APC-Cy7, and anti-CD138-FITC, antibodies for fluorescence-activated cell sorter (FACS) analysis.

Cellular assays

Buffy coat cells were cultured at a concentration of 2.5 x 10^5 cells/mL in supplemented RPMI-1640 media and incubated with 0, 10 nM, 100 nM, and 1000 nM venetoclax with or without 500 nM dexamethasone for 24 h.

Apoptosis Assays

Cell death was measured by flow cytometry after staining with anti-CD38-PE, anti-CD45-APC-Cy7, and Annexin-V-FITC. In samples from patients recently treated with Daratumamab cells were stained with multi-epitope anti-CD38-FITC, anti-CD45-APC-Cy7, and Annexin-V-Pacific Blue. Data were acquired on a BD FACS Canto II flow cytometer and analyzed using BD FACS Diva software (BD Biosciences, San Jose, CA).

CoMMpass analysis

Data for the CoMMpass study (NCT01454297) was downloaded from Genomic Data Commons and processed as previously described(1). Briefly copy number alterations were determined for whole genome sequencing CD138+ myeloma cells relative to CD138- constitutional control (normal) using tCoNut (https://github.com/tgen/MMRF_CoMMpass). *MCL1* expression was determined from

RNA-seq using HTseq. All NDMM patients with both whole genome sequencing and RNA-seq were included.

Immunohistochemistry

Formalin-fixed and paraffin-embedded tissue sections were cut to a 5-µm thickness and air-dried. Staining was performed using Ventana DISCOVERY Ultra automated immunohistochemistry stainer (Ventana Medical Systems, Tuscon, AZ). Slides were deparaffinized with EZ-Prep(# 05279771001,Ventana) and then were antigen retrieved for 64 minutes with CC1 reagent (#950-500, Ventana). Ventana Inhibitor CM was applied to block for endogenous peroxidase. Mouse anti-CD138 antibody (Invitrogen, #MA5-12400) diluted at 1:200 was applied and incubated for 40 min. DISCOVERY OmniMap anti-Mouse HRP was applied and incubated for 12 min and the detection was completed in combination with DISCOVERY ChromoMap DAB kit, as per manufacturer recommendations. Cell Conditioning 2 buffer (CC2, #950-123, Ventana) was used for the denaturation step to remove any unbound CD138. Mouse anti-BCL2 antibody (Cell Signaling, #15071) diluted to 1:200 was applied and incubated for 40 minutes. Discovery Anti-Mouse NP was applied for 8 minutes followed by Anti-NP AP for 8 minutes. The Ventana Discovery Red Alkaline Phosphatase Kit was used per manufacturers recommendations. Slides were counterstained with hematoxylin for 8 min. Slides were then dehydrated, cover-slipped, and evaluated by light microscopy.

BCL2 IHC scoring

BCL2 immunoreactivity (red chromogen cytoplasmic pattern) was scored in CD138 positive plasma cells (brown chromogen membrane pattern). BCL2 immunostaining was semi-quantified into three categories: low reactivity (all or nearly all cells reactive with weak staining), intermediate reactivity (a gaussian like distribution of staining strength from weak to strong), and high reactivity (all or nearly all cells with strong staining). Scoring was performed independently by two pathologists (DLJ, CWS) without prior knowledge of clinical outcomes. For discrepancies, a consensus score was determined by group review of slides.

Statistical Analysis

Genetic risk-stratification was done in accordance with the IMWG definition. Response assessment and progression were evaluated per IMWG Uniform Response Criteria. The IMWG criteria for the definition of sCR was based on attaining CR (negative serum and urine immunofixation, disappearance of soft tissue plasmacytomas and less than 5% plasma cells in bone marrow) plus normalization of sFLC ratio, and absence of clonal plasma cells in bone marrow by immunohistochemistry. Chi-square test and Fisher's exact tests were used while comparing differences between categorical variables, and the non-parametric Mann–Whitney test was used for continuous variables. Survival projections of PFS and OS were estimated by the Kaplan–Meier method and compared by log-rank tests. The Cox proportional hazards model was used to assess for predictors of PFS and OS. Only selected variables reaching statistical significance of p-value <0.05 on univariate analysis were included in the multivariate analysis for both PFS and OS. PFS was calculated as time from venetoclax initiation to

disease progression or mortality from any cause. OS was defined as the time from venetoclax initiation to the date of death or last follow-up.

RNA-seq

50,000 cells were FACS isolated directly into 600μL of RLT lysis buffer with 1% β-ME (Sigma-Aldrich) prior to vortexing 1 min and freezing. RNA was isolated (Qiagen RNeasy Mini) and quality assessed using an Agilent BioAnalyzer. Stranded mRNA-seq libraries were made using the mRNA HyperPrep kit (Kapa) and custom short TruSeq compatible sequencing adapters (IDT) (supplemental table 7).(2) Barcodes were added to each library using long PCR primers (supplemental table 7) during 8 cycles of PCR amplification. Samples were sequenced on a HiSeq 4000 (Illumina).

RNA-seq processing

FASTQ sequencing files were quality and adapter trimmed using Trim Galore! (v0.6.4; https://www.bioinformatics.babraham.ac.uk/projects/trim_galore/) and CutAdapt (v2.5; https://github.com/marcelm/cutadapt/) prior to mapping the GRCh37 genome accounting for the Ensembl GRCh37.74 transcription database using the STAR aligner (v2.5.3a).(3) Putative PCR duplicate reads were marked in BAM files using Samtools (v1.7).(4) Gene read counts were calculated in R (v3.6.2)(5) by reading in BAM files using the function readGAlignmentPairs and counting the number of reads that overlap any exonic region of an Ensemble (v74) gene using the function summarizeOverlaps of the GenomicAlignments package (v1.2).(6) Gene expression data was normalized for sequencing depth using reads per million (RPM) or fragments per kilobase per million

reads (FPKM) where read depth was the total number of reads on autosomal chromosomes only. Data were deposited in GEO and are publicly available, accession GSE167969.

CD20 flow cytometry

CD20 expression was measured by flow cytometry using 4-color panels in red cell depleted bone marrow aspirates as previously described (7). Samples were considered positive if population florescence was above the internal control population in the same tube.

References

1. Barwick BG, Neri P, Bahlis NJ, Nooka AK, Dhodapkar MV, Jaye DL, et al. Multiple myeloma immunoglobulin lambda translocations portend poor prognosis. Nature communications. 2019;10(1):1911.
2. Bowman SK, Simon MD, Deaton AM, Tolstorukov M, Borowsky ML, Kingston RE. Multiplexed Illumina sequencing libraries from picogram quantities of DNA. BMC genomics. 2013;14:466.
3. Dobin A, Davis CA, Schlesinger F, Drenkow J, Zaleski C, Jha S, et al. STAR: ultrafast universal RNA-seq aligner. Bioinformatics (Oxford, England). 2013;29(1):15-21.
4. Li H, Handsaker B, Wysoker A, Fennell T, Ruan J, Homer N, et al. The Sequence Alignment/Map format and SAMtools. Bioinformatics (Oxford, England). 2009;25(16):2078-9.
5. Gentleman RC, Carey VJ, Bates DM, Bolstad B, Dettling M, Dudoit S, et al. Bioconductor: open software development for computational biology and bioinformatics. Genome biology. 2004;5(10):R80.
6. Lawrence M, Huber W, Pages H, Aboyoun P, Carlson M, Gentleman R, et al. Software for computing and annotating genomic ranges. PLoS computational biology. 2013;9(8):e1003118.
7. Kraus TS, Sillings CN, Saxe DF, Li S, Jaye DL. The role of CD11c expression in the diagnosis of mantle cell lymphoma. Am J Clin Pathol. 2010;134(2):271-7.

Supplemental Figures

Supplemental Figure 1

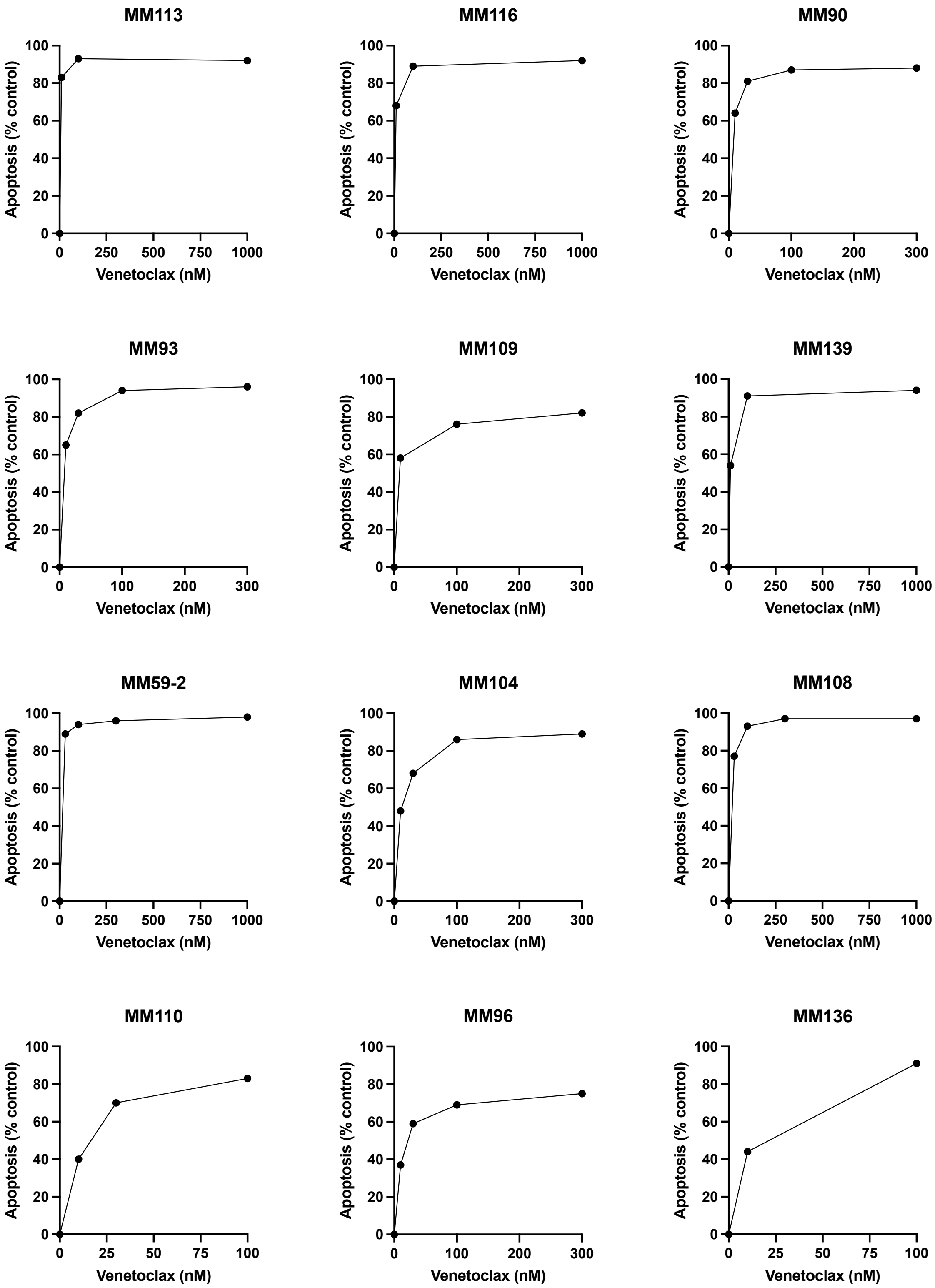

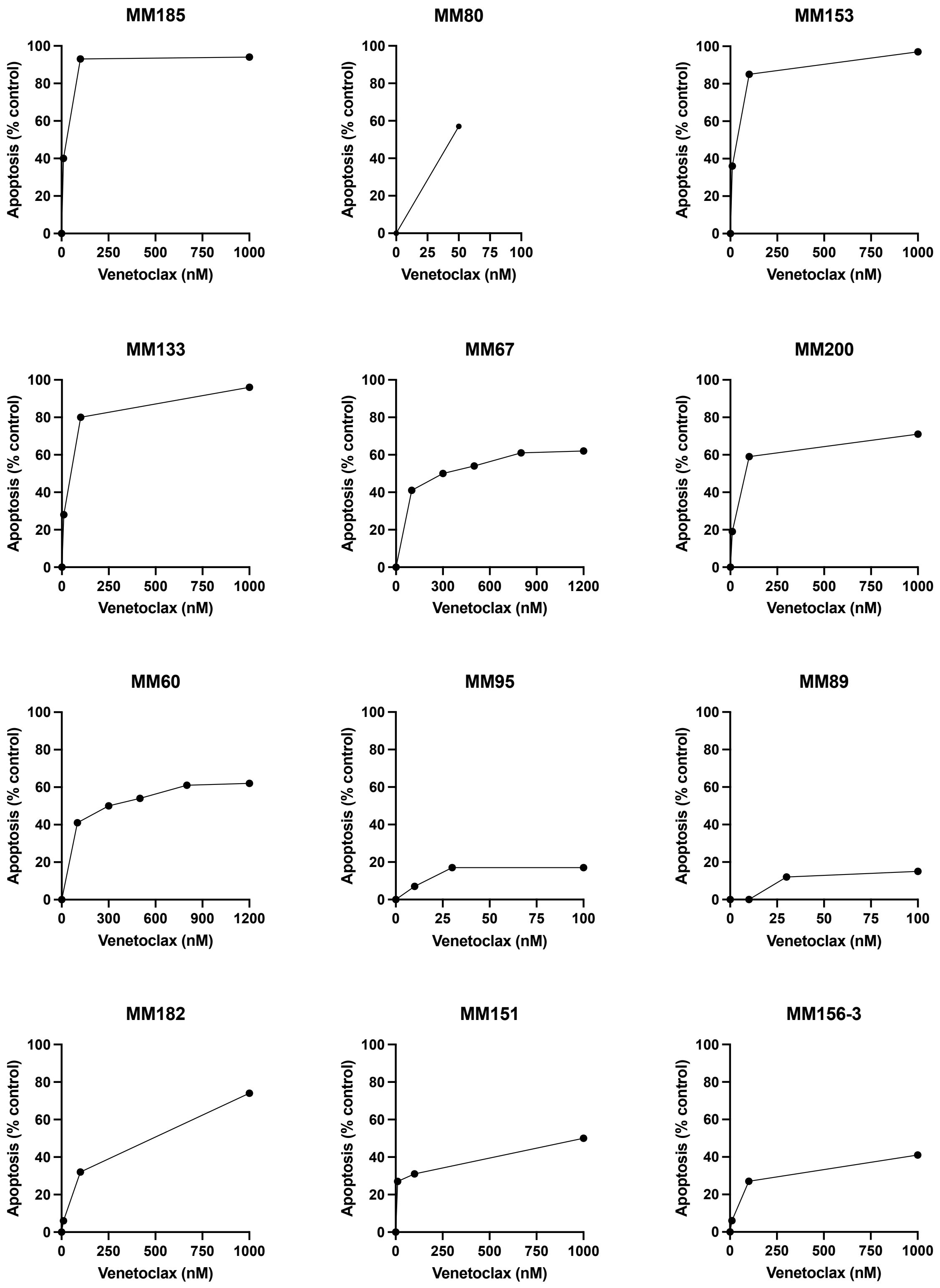
MM185
MM80
MM153
MM133
MM67
MM200
MM60
MM95
MM89
MM182
MM151
MM156-3
Apoptosis (% control)
Venetoclax (nM)

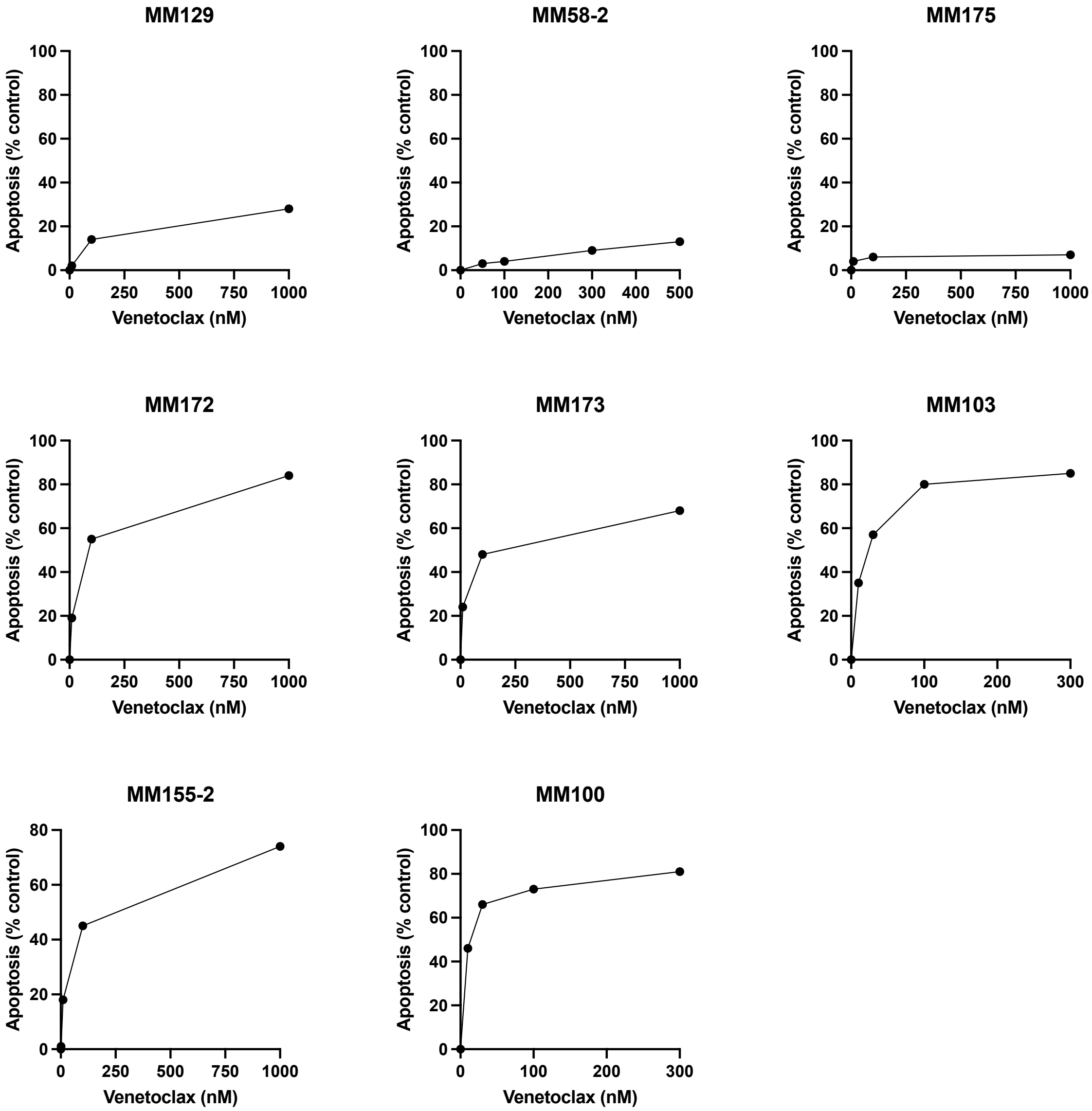

Individual dose curves for patient samples. Samples were treated with indicated doses of venetoclax for 24 hours prior to measuring apoptosis by flow cytometry in order to calculate the IC50. Patients MM172, MM173, MM103, MM155-2, and MM100 were either non-secretory or co-treated with daratumumab or carfilzomib and therefore excluded from analysis.

Supplemental Figure 2

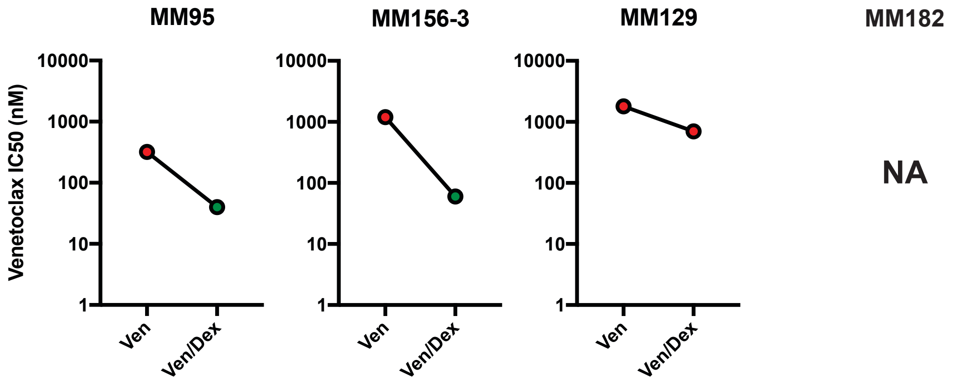

BCL2 IHC High Low High Intermediate

Characterization of the 4 patients resistant to venetoclax ex vivo that ultimately responded to venetoclax treatment. Either addition of dexamethasone or presence of high/intermediate BCL2 by IHC sensitizes myeloma to venetoclax. Three patient samples were treated with increasing concentrations of venetoclax without (ven) and with 0.5 micromolar dexamethasone (ven/dex) to determine an IC50. Green: IC50 < 100 nM. Red: IC50 > 100 nM. BCL2 IHC results are included below the graph.

Supplemental figure 3

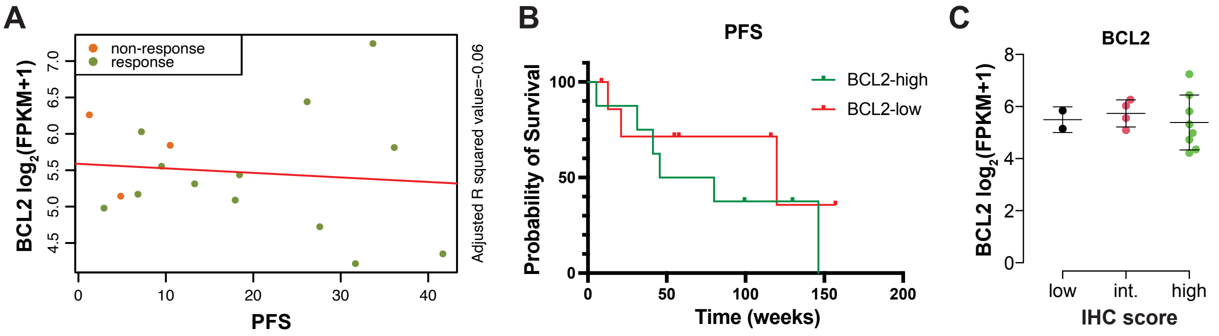

BCL2 RNA expression does not correlate with response or protein expression by IHC. RNAseq data were available for 16 patients. (A) Scatter plot of BCL2 RNA

expression vs. PFS in months. Non-responding patients (n=3) are in orange, responding patients (n=13) are in green. (B) Kaplan-meier curve comparing patients high vs low BCL2 RNA expression. High vs. low was determined by expression greater than or less than the median, respectively. (C) RNA expression compared to BCL2 IHC divided into low (n=2), intermediate (n=4), or high (n=8) staining.

Supplemental figure 4

A

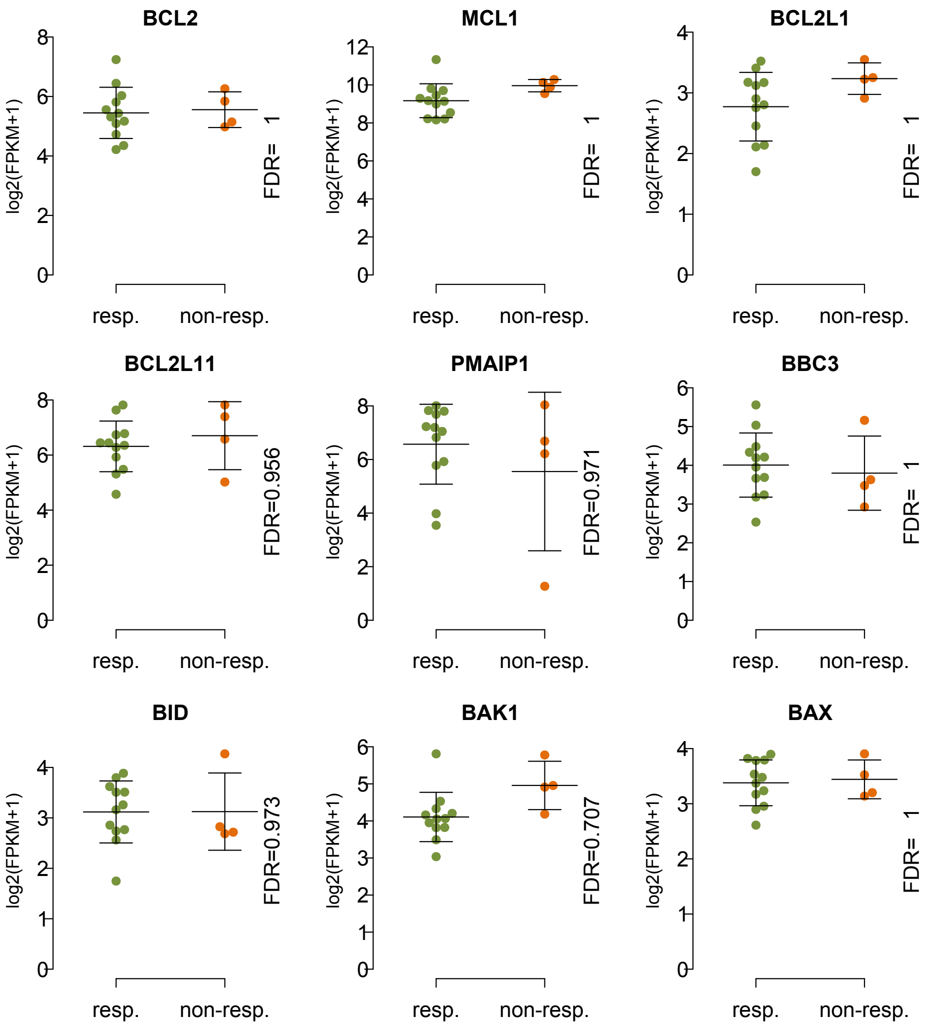

B

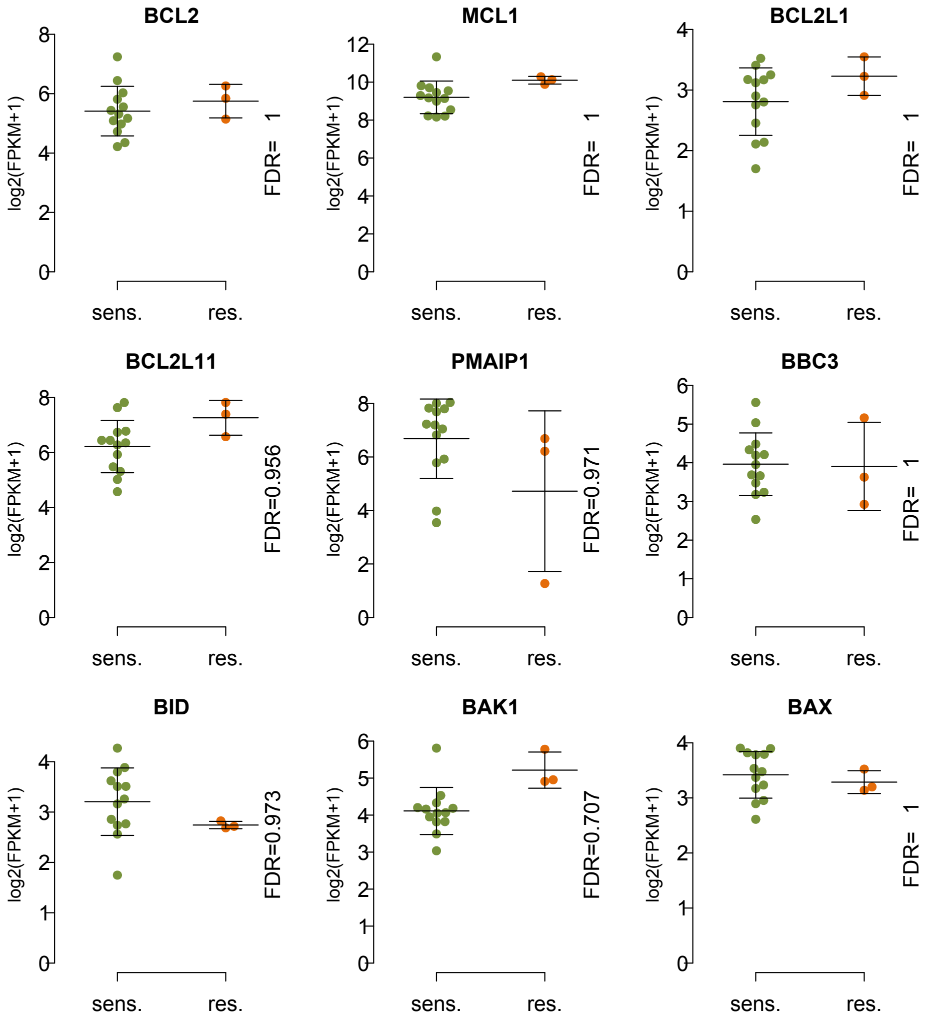

RNA Expression of BCL2 family members do not differ. RNAseq $\log_2$(FPKM+1) expression of indicated BCL2 family members in (A) responders (green, resp) vs. non-responders (orange, non-resp) or (B) sensitive (green, sen) vs. resistant (orange, res) samples

Supplemental figure 5

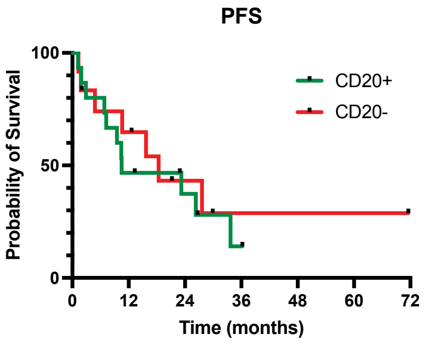

CD20 alone does not predict response to venetoclax. Kaplan-meier curve comparing PFS of patients with (n=15) or without (n=12) CD20 expression by clinical flow cytometry.

Supplemental table 1. Patient information including ex vivo venetoclax IC50, BCL2 IHC staining, venetoclax dose (Ven), use of dexamethasone (dex), clinical response demographics, cytogenetics, and treatment history.

Samples	IC50 (nM)	BCL2 IHC	Ven dose (mg)	Add'l tx	Response	Sex	Age	ISS	FISH	Prior lines	Len ref	Bor ref	Pom ref	Car ref	Dara ref	ASCT
MM113	6	High	800	dex	sCR	F	70	NA	t(11;14)	1	N	N	N	N	N	Y
MM116	7	High	400		sCR	M	51	1	+1q, +11	2	Y	Y	N	N	Y	Y
MM90*	8	High	800	dex	VGPR	M	68	NA	t(11;14), del(13q)	1	Y	Y	N	N	N	Y
MM93*	8	Int	800	dex	PR	F	78	3	t(11;14), del(17p), del(13q)	2	Y	Y	Y	Y	N	N
MM109*	9	High	800		PR	M	79	NA	+3, +11	1	Y	N	N	N	N	Y
MM139	9		800		sCR	M	66	3	t(11;14), del(13q)	1	Y	Y	N	N	N	Y
MM59-2*	16	High	800	dex	CR	M	73	NA	t(11;14)	4	Y	Y	Y	Y	Y	Y
MM104*	18		800	dex	PR	F	72	2	t(11;14), +1q, -13	2	Y	Y	Y	Y	N	Y
MM108*	19	Low	800		sCR	F	48	NA	t(11;14), +1, +3, +7, +9, +13, +17	3	Y	Y	Y	N	Y	Y
MM110	20	Int	800	dex	VGPR	M	56	3	t(11;14), -13, del(17p)	7	Y	Y	Y	Y	Y	Y
MM96*	20	Low	400	dex	SD	M	69	NA	t(11;14), +1q	7	Y	Y	Y	Y	Y	Y
MM103*	24	High	800	dex	VGPR	F	64	2	t(11;14), -1q, -13	2	Y	Y	N	N	N	Y
MM136	40	High	800		sCR	M	60	3	t(11;14)	2	Y	Y	Y	N	N	Y
MM185	40	High	800	dex	PR	F	67	1	t(11;14), +1p, +1q, +3, +7, +9, -13, del(17p)	5	Y	Y	Y	Y	Y	Y
MM80*	40	High	1200		PR	M	60	2	t(11;14)	4	Y	Y	N	N	N	Y
MM153	50	High	800	dex	PR	M	63	2	t(11;14)	3	Y	Y	Y	N	N	Y
MM133	60	Low	800	dex	SD	F	75	2	t(11;14), +1q, -13	3	Y	Y	Y	N	N	Y
MM67*	60	High	900		sCR	F	42	3	t(11;14), +1q, del(13q)	4	Y	Y	Y	N	N	Y
MM200	80		400	dex	sCR	M	72	NA	t(11;14)	5	Y	Y	Y	Y	Y	Y
MM60*	300	Low	600		PD	M	53	2	t(11;14), +1q	2	Y	Y	N	N	N	Y
MM95*	320	High	800	dex	PR	F	63	3	t(11;14), +1q, del(13q)	3	Y	Y	N	N	Y	Y
MM89*	330	Low	800	dex	PD	M	62	1	t(11;14), +1	3	Y	Y	Y	Y	Y	Y
MM182	600	Int	800	dex	VGPR	M	76	2	t(11;14), +1q	3	Y	Y	Y	N	Y	Y
MM151	1000	Low	800	dex	SD	F	63	2	t(11;14), +1p, +1q, +3, del(13q), +17	5	Y	Y	Y	Y	N	Y
MM156-3	1200	Low	800	dex	sCR	M	76	2	t(11;14), +1q, +3	6	Y	Y	Y	Y	Y	Y
MM129	1800	High	800	dex	VGPR	M	65	NA	t(11;14), +1p, +1q, +3, +7, +9, +13 del(17p)	5	Y	Y	Y	Y	Y	Y
MM58-2*	1980	Int	600		PD	M	56	1	t(11;14), del(17p), +1q, del(13q)	5	Y	Y	Y	Y	N	Y
MM175	7000	High	800	dex	SD	M	66	2	t(11;14), +1q, +9	3	Y	Y	N	Y	Y	Y

Supplemental table 2. Univariate analysis of patient variables.

	All Patients (N=28)	Ex-vivo (sens)(N=19)	Ex-vivo (res)(N=9)	P-value
Median age at diagnosis, y	59.8 (38.9-77.3)	60.4 (38.9-77.3)	58.8 (50.2-71.2)	0.225
Median age at start of venetoclax, y	65.2 (42.3-78.8)	67.4 (42.3-78.8)	63 (52.9-76.4)	0.225
Median time from diagnosis to start of venetoclax, m	64.1 (7.2-177.4)	66.2 (7.2-177.4)	58.7 (26.9-149.9)	0.686
Median number of prior lines of therapy	3 (1-7)	3 (1-7)	3 (2-6)	0.7
Male	18 (64%)	11 (58%)	7 (78%)	0.305
Female	10 (36%)	8 (42%)	2 (22%)	
White	24 (86%)	16 (84%)	8 (89%)	0.741
Black	4 (14%)	3 (16%)	1 (11%)	
ISS stage III	6 (30%)	5 (42%)	1 (13%)	0.378
t (11;14)	26 (93%)	17 (90%)	9 (100%)	0.312
1q gain of 1 copy	14 (50%)	5 (26%)	9 (100%)	0.001
1q gain of ≥2 copies	3 (11%)	0 (0%)	3 (33%)	<0.001
Del 17p	8 (29%)	6 (32%)	2 (22%)	0.609
pPCL	1 (4%)	1 (5%)	0 (0%)	0.483
High-risk	9 (32%)	7 (37%)	2 (22%)	0.439
Daratumumab refractory	13 (46%)	7 (37%)	6 (67%)	0.139
Carfilzomib refractory	13 (46%)	7 (37%)	6 (67%)	0.139
Pomalidomide refractory	18 (64%)	12 (63%)	6 (67%)	0.856
Lenalidomide refractory	27 (96%)	18 (95%)	9 (100%)	0.483
Bortezomib refractory	26 (93%)	17 (90%)	9 (100%)	0.312
ASCT	27 (96%)	18 (95%)	9 (100%)	0.483
≥3 lines of therapy	18 (64%)	10 (53%)	8 (89%)	0.06
≥4 lines of therapy	11 (39%)	7 (37%)	4 (44%)	0.7
≥5 lines of therapy	8 (29%)	4 (21%)	4 (44%)	0.201
≥6 lines of therapy	3 (11%)	2 (11%)	1 (11%)	0.963
